# COVID-19 modeling based on real geographic and population data

**DOI:** 10.55730/1300-0144.5589

**Published:** 2022-12-31

**Authors:** Emir BAYSAZAN, A. Nihat BERKER, Hasan MANDAL, Hakan KAYGUSUZ

**Affiliations:** 1 TEBIP High Performers Program, Council of Higher Education, İstanbul University, İstanbul, Turkey; 2 Faculty of Engineering and Natural Sciences, Kadir Has University, İstanbul, Turkey; 3 TÜBİTAK Research Institute for Fundamental Sciences, Kocaeli, Turkey; 4 Department of Physics, Massachusetts Institute of Technology, Cambridge, Massachusetts, USA; 5 The Scientific and Technological Research Council of Türkiye (TÜBİTAK), Ankara, Turkey; 6 Department of Basic Sciences, Faculty of Engineering and Architecture, Altınbaş University, İstanbul, Turkey; 7 SUNUM Nanotechnology Research Center, Sabancı University, İstanbul, Turkey

**Keywords:** Monte Carlo simulation, epidemic, geographical model, susceptible-infected-quarantine-recovered model, COVID-19

## Abstract

**Background/aim:**

Intercity travel is one of the most important parameters for combating a pandemic. The ongoing COVID-19 pandemic has resulted in different computational studies involving intercity connections. In this study, the effects of intercity connections during an epidemic such as COVID-19 are evaluated using a new network model.

**Materials and methods:**

This model considers the actual geographic neighborhood and population density data. This new model is applied to actual Turkish data by means of provincial connections and populations. A Monte Carlo algorithm with a hybrid lattice model is applied to a lattice with 8802 data points.

**Results:**

Around Monte Carlo step 70, the number of active cases in Türkiye reaches up to 8.0% of the total population, which is followed by a second wave at around Monte Carlo step 100. The number of active cases vanishes around Monte Carlo step 160. Starting with İstanbul, the epidemic quickly expands between steps 60 and 100. Simulation results fit the actual mortality data in Türkiye.

**Conclusion:**

This model is quantitatively very efficient in modeling real-world COVID-19 epidemic data based on populations and geographical intercity connections, by means of estimating the number of deaths, disease spread, and epidemic termination.

## 1. Introduction

The coronavirus disease 2019 (COVID-19) pandemic is caused by the severe acute respiratory syndrome coronavirus 2 (SARS-CoV-2). The use of several vaccination measures, such as CoronaVac (Sinovac Biotech, China), Sputnik V (Gamaleya Research Institute of Epidemiology and Microbiology, Russia), Comirnaty (Pfizer-BioNTech, Germany), and Turkovac (Health Institutes of Türkiye and Erciyes University, Türkiye), has reduced the effects of the pandemic [1]. However, it is still considered to be ongoing and has caused societal impacts, including in urban areas. The complexity of the pandemic has been compounded by the interconnected nature of societal activities.

During the pandemic, indication to vaccine development [1], many countries adapted different methods to combat this disease. These include improved hygiene measures, vaccination, curfews, and full quarantines. Türkiye is among the countries which applied full quarantines followed by curfews on weekends. Among the precautions, intercity travel was restricted and subject to special permission as one of the measures to combat disease transmission given the spatial diffusion patterns through intercity mobility [2].

Many scientists and research groups conduct their studies using in silico methods (experimental simulations performed on computers) of genetic analysis [3,4]. Computation methods also include the epidemiological simulation. Two main approaches followed in the literature are fitting the clinical data with various mathematical models and the simulation of the epidemic with various mathematical models [5]. Previously, several studies on modeling infectious diseases have been reported [6–10]. Among the simulation methods, Monte Carlo (MC) simulation is an effective method [11]. Currently, various MC studies are present for COVID-19 simulation. These include analyzing different scenarios for selected countries [12], age-structured mobility data for simulation of the pandemic spread in selected cities [13], random-walk proximity-based infection spread [14], and observing the effect of weekend curfews [15].

Travel restrictions are among the first emergency measures [16] during epidemics. Intercity travel models on epidemic spreading can be modeled using the well-known small-world networks [17] and recent papers report such simulations [18]. Big data analytics have been used to compare mobility patterns during the pandemic in Finland [19] while other studies have focused on the impacts of the pandemic on public transport [20]. Moreover, the most recent report of the Intergovernmental Panel on Climate Change underlined the impacts of the pandemic on society, including the transport sector [21].

This paper discusses the effects of intercity travel during a pandemic, such as COVID-19, using a new model network on a selected country (Türkiye) by considering the actual geographic neighborhood and population density data. The spread of the disease was modeled using a hybrid of susceptible, infected, quarantine, and recovered (SIQR) [22–24] lattice model [25] and spin-1 Ising model [26,27], which was previously reported in our recent paper [15]. The present study addresses the gap in the literature for coupling actual geographic neighborhood and population density data with a hybrid lattice model.

## 2. Methods

**Figure 1:**
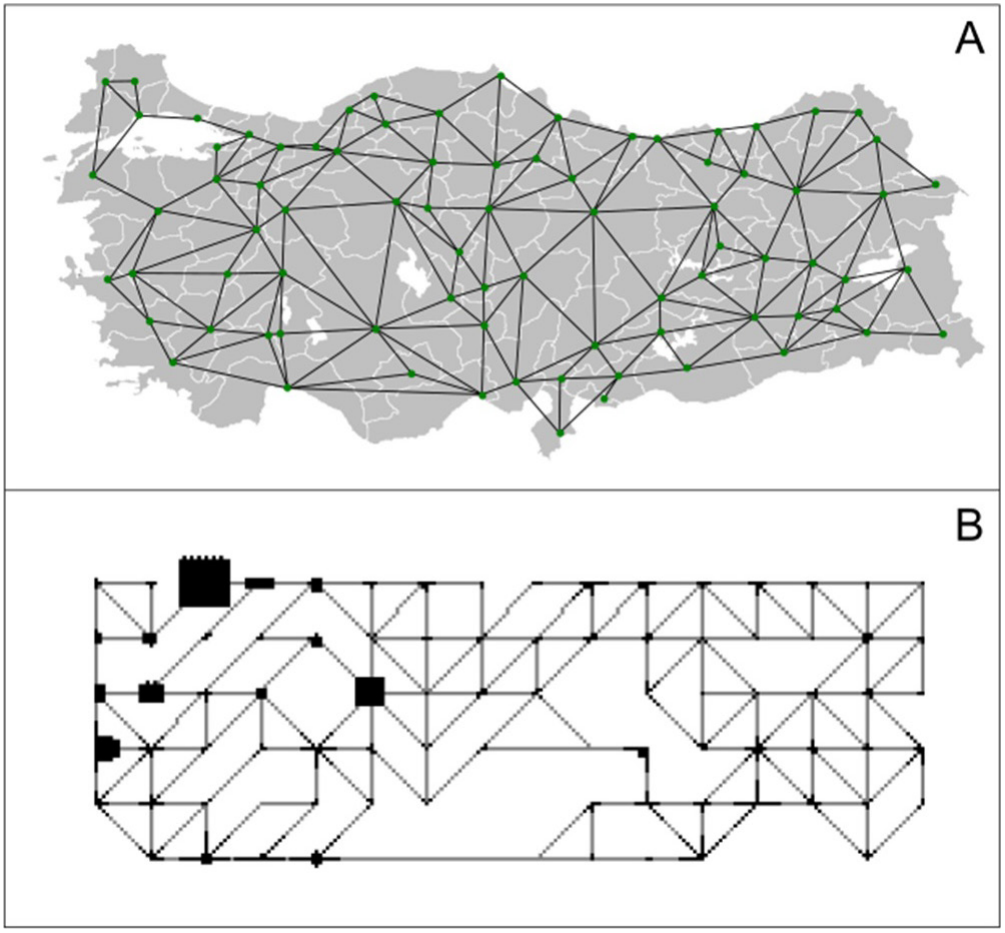
Lattice model based on the provincial neighborhoods overlaid on the map of Türkiye (A) and population weighted lattice of the same lattice (B).

The MC model used in this study is written in the Python language by the authors. A lattice model with 8802 data points was generated using the map of Türkiye, taking into consideration the neighborhoods of provincial centers and population densities. Figure 1A shows the lattice model based on the provincial centers of Türkiye and Figure 1B indicates the population-weighted version of the one-to-one same lattice, where provinces with more population are represented with bigger lattice blocks. The lattice shown in Figure 1B is considered in further simulations.

Provinces with bigger population are shown in bigger lattice blocks, where the actual population data was obtained from the Turkish Statistical Institute.^1^ The network is generated on an Ising-like model where the persons are placed on lattice points. The number of people in each block (city) in the matrix has been assigned according to their ratio in the total Turkish population. On the border of each town, there are roads that provide intercity connections. These are designed as connections between square-shaped population blocks. The connections are unpopulated; however, they can transmit the disease to a person in the neighboring town. For each person in the network, five different states are possible: Healthy (susceptible), positive, sick (under quarantine), recovered, and deceased. Each person is represented in the lattice as integers, and the cases mentioned above are assigned as 1, 2, 3, 4, and 5 (deceased), respectively. In the algorithm, calculations and decisions are made based on the values of each data point. For example, if the value of the lattice point is 3, the movement of this particle is neglected since this case is considered to be quarantine. Unpopulated parts in the total lattice are assigned as forbidden lattice points.

For the MC simulation, persons in the lattices are randomly selected and assigned a random possible direction for the movement. Firstly, neighbors (i.e. side neighbors including intercity connections and corner neighbors on the square lattice) are checked for contamination with probability *P*. Secondly, the selected person moves in the selected direction with probability *P*. If the selected direction contains a person, this happens by person interchange.

One MC step in this simulation is defined as considering all particles in the lattice for infection and movement. The simulation considers the following scenario: At step 0, a random person gets positive (patient zero) in İstanbul (since it is the largest city with arguably the largest cosmopolitan connections) and starts spreading the disease. Once a person gets into contact with the virus, the person can remain in quarantine with a probability of *P_quarantine_* = 0.33 or be recovered with a probability of *P_recovery_* = 0.33 at each MC Step. A sick person under quarantine cannot move and cannot spread the disease, removing the probability of movement and infection for this lattice points. Persons under quarantine recover with a probability of *P_treatment_ =* 0.995 or is deceased with probability 1-*P_treatment_*, after 7 MC steps. This ratio is arranged according to total mortality rate of COVID-19 in Türkiye (approximately 0.005). For each person, a random number is generated in the script and if this random number is bigger than the probability *P*, the related event occurs. In this model, no lockdowns are considered.

## 3. Results

**Figure 2:**
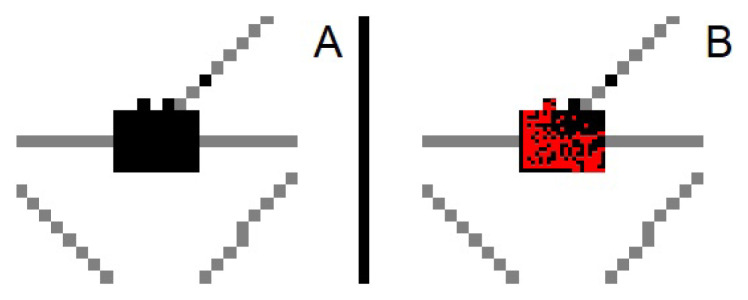
Spread of the disease in Bursa a) day 0, b) day 50.

Figure 2 shows the virus spread in Bursa on days 0 and 50, respectively. Persons with the disease are marked with red.

The result of the epidemic is tested using the susceptible, infected, in quarantine, and recovered (SIQR) model, which was previously applied for COVID-19 [28].

**Figure 3:**
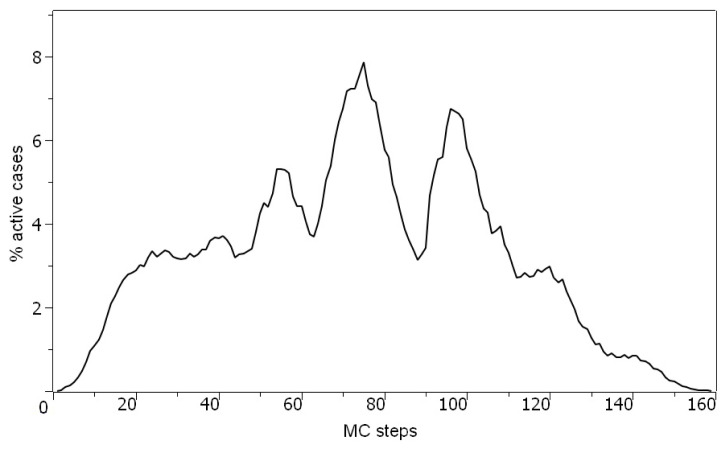
Number of active cases (as a percentage of population) in this model.

Figure 3 shows the active positive cases in the simulation. Around MC step 70, the number of active cases in Türkiye reaches up to 8.0% of the total population, followed by a second wave around MC step 100, around 7.0%. After this second wave, the number of active cases decreases and reaches down to 0 around MC step 160.

**Figure 4:**
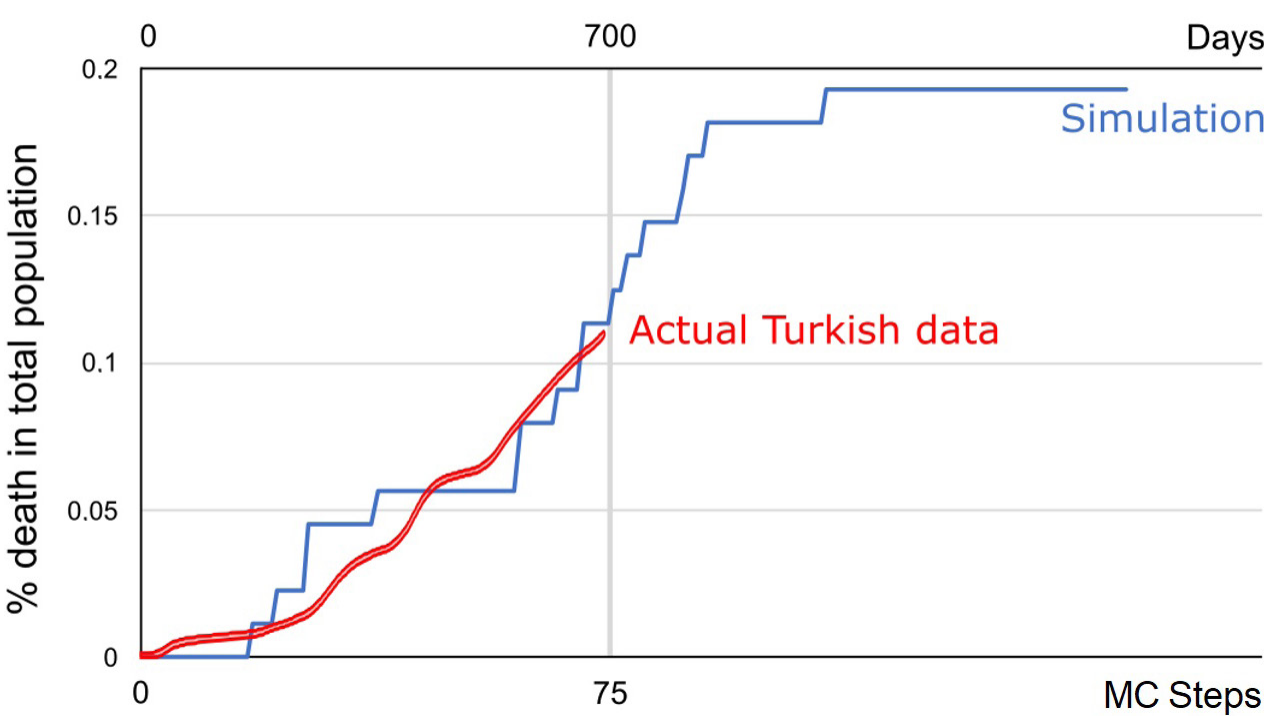
Percentage of deaths due to COVID-19, simulation compared with real Turkish data.

Among the different epidemic measures, the primary goal is to reduce the number of deaths. The number of deaths for Türkiye from the beginning of the pandemic is compared with the simulation data and is shown in Figure 4, by means of day-percentage of death to the total population. Real-world data was obtained from Worldometer.^2^

**Figure 5:**
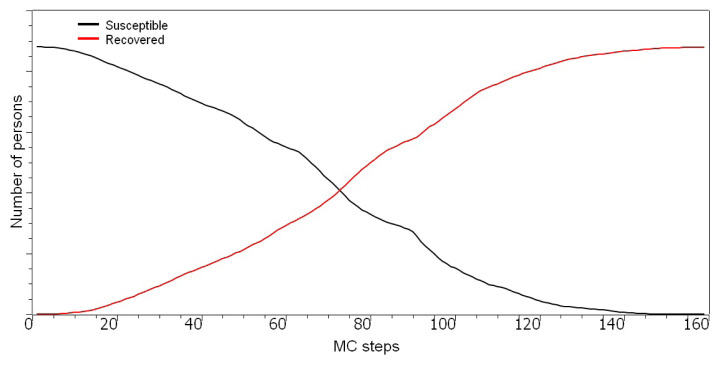
Number of healthy (susceptible) and recovered individuals over MC steps.

Results show that the simulation fits real-world data well and can be used as an efficient tool to predict the number of deaths. Another important outcome is that ten MC steps of the simulation are around one real-time day. This implies that the population density-based lattice of Turkish provinces is effective in modeling the real data. Numbers of healthy and susceptible individuals are also provided in Figure 5.

**Figure 6:**
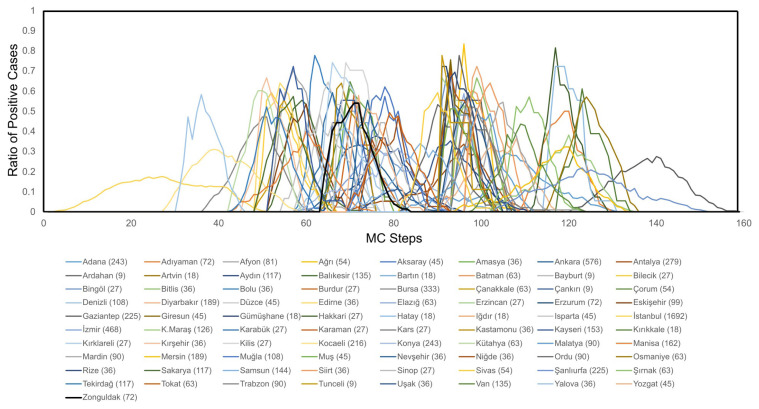
Spread of the epidemic in 81 Turkish provinces (names and number of persons in each province are shown below the plot).

**Figure 7:**
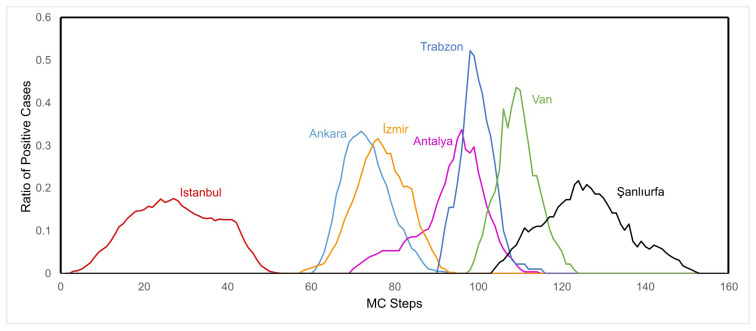
Spread of the epidemic in seven cities of seven geographical regions of Türkiye.

Another subject examined in this study is the spread of diseases in different provinces of Türkiye. In order to visualize the spread of the disease, the percentage of positive cases (quarantined and nonquarantined) in the province was evaluated for each block (province) and the results for 81 Turkish provinces are shown in Figure 6. Starting with İstanbul, the epidemic quickly expands into most provinces between MC steps 60 and 100. Seven selected provinces from seven geographical regions of Türkiye are also examined for highlighting the spread of epidemic in different regions (in Figure 7). It is shown that the spread vanishes at the Southeastern Anatolia Region around MC step 160.

## 4. Discussion

In this model, the infectivity and the mortality of the virus are constant, which means there are no variants of the initial virus. In addition, vaccination is neglected, which assumes that the entire population has the same level of immunity to the virus. Under these circumstances, the epidemic in Türkiye extinguishes on 160 MC steps. Currently, the pandemic continues, but daily active cases are about to run out around the date the article is written.

COVID-19 epidemic simulations at the local and national level are among the important contributions to the fight against the pandemic. A recent study which simulates the COVID-19 outbreak in Bogotá, Colombia uses an extension of a compartmental susceptible-exposed-infected-recovered model with random perturbations and is found to be robust in projecting the numbers in this case [29]. Resurgence of COVID-19 in Chinese local communities were also studied using a similar method [30,31]. Another recent study revealed the results of a spatio-temporal simulation based on big data and gravity model in Guangzhou, China, where the simulation is able to identify different transmission patterns which are dependent on the urban spatial structure [31]. Although obtaining real data for these kinds of studies is a limitation in the beginning, all of these studies are found to be important for policymakers to make reliable and fast interventions during combating the pandemic.

Another significance of this study is that it presents an approach to the modeling of geographic connections and information such as population. In the case of Turkey, besides the fact that the epidemic occurred in large centers such as Ankara, İstanbul, and İzmir, its impact on smaller cities in terms of population has also been discussed. In a more detailed analysis, adding districts and even rural centers to the design will probably give a more accurate result, but this time there will be additional requirements to construct such a geographic model in a 2-dimensional matrix. As a future study, transportation connections, population density, and even working hours in a selected settlement (district) can be included in the simulation.

In conclusion, in this study, a novel approach to COVID-19 epidemic simulation was conducted and tested on epidemic data in Türkiye. This model uses the provincial neighborhood and population data. A hybrid MC algorithm ensured a very efficient modeling of the epidemic by means of growth time and number of deaths. Further studies can reveal this model in different countries, as well as the effects of different prevention measures, such as different types of vaccines, travel bans, and curfews. The coupling of spatiotemporal datasets with the hybrid lattice model is promising of numerous other advances in the field.

## Footnotes

1 Turkish Statistical Institute (2021). Address Based Population Registration System Results [online]. Website https://data.tuik.gov.tr/Bulten/Index?p=Adrese-Dayali-Nufus-Kayit-Sistemi-Sonuclari-2021-45500 [accessed 14 May 2022].

2 Worldometer (2022). Turkey COVID - Coronavirus Statistics [online]. Website https://www.worldometers.info/coronavirus/country/turkey [accessed 14 May 2022].
